# The Plans of the Sheffield Royal Hospital

**Published:** 1912-05-25

**Authors:** 


### The Plans of the Sheffield Royal Hospital.

*To the Editor of* The Hospital.

Sir,—I have followed with interest from the architectural standpoint the articles in *The Builder* and The Hospital in reference to the above. While, I am sure, every right-thinking architect welcomes criticism of his work, and particularly so from an acknowledged hospital authority such as Sir Henry Burdett, I should like to point out that frequently the architect's sphere is limited both by finance and an unintelligent committee. Both are obstacles which may be deplored but still must be faced, and it therefore seems right that the investigation committee in this particular case should publish not only the plans of the completed work but also the architect's original reports to the authorities; for it will prove interesting reading to learn in how far the architect was overridden in his recommendations. Your article is somewhat misleading on the subject of the ownership of drawings; they are usually reputed to be the property of the architect and not of the employer, and this view has, I think, been upheld by a test case. I enclose my card and remain

Yours, etc.,

Audi Alteram Partem.

